# *Cryptosporidium* and *Giardia* infections of lambs in Southwest Norway: a longitudinal study

**DOI:** 10.1186/s13028-025-00823-8

**Published:** 2025-08-13

**Authors:** Tsegabirhan Kifleyohannes, Elin Skorpen, Kine Rosnes Hansen, Snorre Stuen, Lucy J. Robertson

**Affiliations:** 1 Parasitology, Department of Paraclinical Sciences, Faculty of Veterinary Medicine, Norwegian University of Life Sciences, Ås, 1433 Norway; 2 Department of Analysis and Diagnostics, Virology, Immunology and Parasitology, Norwegian Veterinary Institute, Ås, 1433 Norway; 3 Skullerudbakken 4J, Oslo, 1189 Norway; 4 Surnadalsvegen 735, Surnadal, 6650 Norway; 5 Department of Production Animal Clinical Sciences, Faculty of Veterinary Medicine, Norwegian University of Life Sciences, Svebastadveien 112, Sandnes, 4325 Norway

**Keywords:** *Cryptosporidium parvum*, *Cryptosporidium ubiquitum*, *Giardia bovis*, Persistent infection, Reinfection, Sheep, Zoonotic potential

## Abstract

**Background:**

Domestic ruminants are common hosts of *Cryptosporidium* spp. and *Giardia* spp. Although both protozoan parasites are known to circulate among lambs in Norway, their epidemiology is largely unknown. This longitudinal study investigated the occurrence of both parasites in different age groups of lambs, with molecular characterisation of some isolates. Faecal samples (*n* = 394) were collected from lambs from 17 different flocks on three occasions. At first sampling, lambs were approximately 2–3 days old, and 160 samples were collected. On the two subsequent occasions, no additional lambs were included and samples collected were from among the lambs sampled on the first occasion. At second sampling, lambs were 14–21 days old, and 134 samples were collected. At the final sampling, lambs were 40–42 days old, and 100 samples were collected. Samples were analysed using immunofluorescent antibody staining (IFAT), with molecular characterisation of selected positive samples by PCR and sequencing.

**Results:**

In total, 66 samples (17%) were positive for *Cryptosporidium* oocysts and 61 (16%) positive for *Giardia* cysts by IFAT. *Cryptosporidium* was detected most often at the second sampling, occurring significantly more often than in younger lambs (*P* < 0.0001). Longitudinal investigation indicated that around 20% of lambs shedding *Cryptosporidium* oocysts at one sampling occasion were still infected, or had been reinfected, at the next sampling occasion. A significant increase in *Giardia* infection occurred with age, with a higher occurrence at the second sampling occasion than the first (*P* < 0.0001), and a greater occurrence at the third sampling occasion than both the first (*P* < 0.0001) and second (*P* = 0.052). For *Giardia*, persistent infection, or reinfection between sampling occasions, was between 25 and 40%. Associations were detected between infection status and location (Vestland or Rogaland), infection status and diarrhoea, and intensity of infection and diarrhoea. Molecular methods identified two species of *Cryptosporidium* (*C. parvum* (subtypes IIaA13G1R2 (4 samples) and IIdA20G1 (1 sample)) and *C. ubiquitum*, subtype XIIa (3 samples). *Giardia* isolates were identified as *G. bovis* (*G. duodenalis*, Assemblage E).

**Conclusions:**

*Cryptosporidium* and *Giardia* infections occurred commonly in lambs in the three different age groups, with more positives detected at the second and third sampling (when the lambs were older) than at the first. As some lambs were positive for one or other of the parasites on two sampling occasions, prolonged infection or reinfection may occur. Molecular characterisation indicated that although the *Cryptosporidium* in these lambs can be of public health importance, the *Giardia* species identified are not considered zoonotic.

**Supplementary Information:**

The online version contains supplementary material available at 10.1186/s13028-025-00823-8.

## Background

*Cryptosporidium* spp. and *Giardia* spp. are ubiquitous protozoan parasites. At least 44 *Cryptosporidium* species and over 120 genotypes, have been identified, of which three are thought to occur commonly in sheep (*C. parvum*,* C. xiaoi*,* C. ubiquitum -* two of which, *C. parvum* and *C. ubiquitum*, are zoonotic), although other species have been identified [1, 2]. Previously sheep were considered to be infected with *Giardia duodenalis*, which was divided into different morphologically identical, but genetically distinct assemblages. However, recent taxonomic revision, which has redescribed these assemblages as species, indicates that sheep are most often infected with non-zoonotic *G. bovis* (previously assemblage E), but may also be infected with other species, some of which have zoonotic potential [3]. Both parasites have been associated with diarrhoeal disease, cryptosporidiosis and giardiosis, both of which have been documented in ruminants globally [4], with younger animals most frequently affected.

Infection in neonatal and young ruminants with *Cryptosporidium* and *Giardia* is both a welfare issue and an economic burden for farmers; not only do these infections cause diarrhoeal illness and failure to thrive in young ruminants, with cryptosporidiosis sometimes being fatal, but also stunt growth and reduce productivity [4, 5, 6].

A longitudinal study of lambs from six flocks in Norway in 2008 [7] revealed the occurrence of both potentially zoonotic *Cryptosporidium* species (cervine genotype, now known as *C. ubiquitum*) and non-zoonotic species (*C. xiaoi*), but *C. parvum*, which is considered to have greatest zoonotic potential, was not identified. In the same study, the majority of *Giardia* isolates analysed (41/42) were non-zoonotic *G. bovis* (syn. *G. duodenalis* assemblage E), with one isolate identified as zoonotic assemblage B (now known as *G. enterica*).

Based on these results, the authors suggested that sheep in Norway are unlikely to be an important reservoir of zoonotic *Giardia* [7] but might have some public health significance with respect to *Cryptosporidium*. Indeed, human outbreaks of cryptosporidiosis associated with contact with lambs have been reported from Norway [8], as well as other countries [9], and an outbreak of cryptosporidiosis (infection with *C. parvum*, subtype IIdA23G1) in veterinary students in Norway occurred in spring 2024 associated with lambing activities [10].

However, despite these outbreaks, it has been more than a decade since investigations on *Cryptosporidium* and *Giardia* on lambs in Norway were conducted. Information regarding the current status of *Cryptosporidium* and *Giardia* circulating in lambs in Norway, and insights on the epidemiology of these infections in small ruminants in Norway are lacking. This study was initiated to investigate the occurrence of *Cryptosporidium* and *Giardia* in different age groups of lambs using a longitudinal approach in which the same lambs were sampled on up to 3 occasions to determine the age where these infections predominated, and to elucidate whether there was an age-related association with different species or genotypes of parasites. In addition, by identifying the species of *Cryptosporidium* and *Giardia* in lamb infections, we wished to investigate further the zoonotic potential.

## Methods

### Study areas

A longitudinal study design was used to investigate the occurrence of *Cryptosporidium* and *Giardia* in different age groups of lambs from 17 farms in two regions in two separate counties in the southwest of Norway: Sunnhordland in Vestland (*n* = 11) and Dalane in Rogaland (*n* = 6) (Additional file 1).

Both counties have large populations of sheep; Rogaland is the county with the highest winter-fed sheep population in Norway (around 190,000) and Vestland has a population of around 178,000 winter-fed sheep [11]. The climate in these counties is relatively mild, being cool and damp; as the parasite transmission stages survive well in such environments, there is potentially higher parasite infection pressure here than elsewhere in Norway where the climate is often harsher.

Of the farms included in this study, those included in the study in Vestland had, on average, 124 sheep (range: 27–300) and those from Rogaland had on average 171 sheep (range: 60–350). Sheep breeds were predominantly Norwegian White Sheep (the most common breed farmed in Norway) and Norwegian Short Tail Landrace.

### Target population

This was a longitudinal study, in which samples were collected on three occasions from the same lambs (lambs sampled on the 2nd and 3rd occasions had been sampled on the first occasion). Due to pragmatic reasons, sampling was based upon convenience and cooperation with farmers, rather than a pre-determined sampling plan. An approximate sample size of 100 samples per sampling occasion was considered sufficient based on results obtained from the previous investigation of these parasites in Norway [7] in which around 100 samples per sampling occasion were collected per farm, and it was possible to determine parasite species and occurrence. For pragmatic reasons, it was not possible to analyse 100 samples per farm in the current study; this is a clear limitation of the study.

At the first sampling, lambs were 2–3 days old, at the 2nd sampling 2–3 weeks old, and at the final sampling they were around 6 weeks old. These sampling time points were chosen within the available sampling period to cover the most likely earliest shedding of *Cryptosporidium* oocysts from infected lambs [12] until around 6 weeks later. Both male and female lambs were included. Bottle-fed lambs were excluded from the study. Due to difficulties with tracking the lambs, the number of lambs sampled on each occasion decreased with time. That is, not all lambs that were sampled on the 1st occasion were sampled on the subsequent occasions, with an overall drop of around 38% from 160 samples on the first occasion to 100 samples on the last occasion. After the first sampling, no further lambs were added to the study.

### Collection of samples

Faecal samples were collected directly from the rectum of lambs from March to May 2021 on the three sampling occasions. The lambs were all superficially healthy and although they had been treated prophylactically/metaphylactically for helminths and/or *Eimeria* according to the farmers routines they had not, to our knowledge, been treated with halofuginone or paromomycin sulphate. Samples were transferred into small, labelled plastic bags and stored at 4 °C until laboratory processing, as soon as possible after collection (within 3 weeks of collection).

### Detection of *Cryptosporidium* oocysts and *Giardia* cysts

For pragmatic and economic reasons, samples from siblings that were non-diarrhoeic were merged and examined as a combined sample. If parasites were not detected in the combined sample, then the results were recorded as negative for both lambs (two negative samples). If the sample was positive, then the individual samples were examined separately to determine which lamb, or both, were shedding parasites. Diarrhoeic samples were not combined for screening.

Approximately 20 µL of hand-homogenised faecal sample was smeared on a microscope slide using a disposable bacteriological loop. This was air dried at room temperature and fixed with a drop of methanol. Fixed slides were stained with FITC-conjugated monoclonal antibodies (mAbs) against *Cryptosporidium* oocyst walls and *Giardia* cyst walls (Aqua-Glo, Waterborne Inc., NO, USA) and then stained with 4´, 6- diamidino-2-phenylindole (DAPI) to visualise nuclei. Slides were screened by fluorescence microscopy using a Leica DCMB microscope (200x and 400x magnification) using filter blocks appropriate for FITC (blue – emission 490, excitation 525) and DAPI (UV - emission 350, excitation 470).

The samples were scored based on the detection of *Cryptosporidium* and *Giardia* (oo)cysts. The results were either classified as negative (no identification of (oo)cysts) or scored by the average number of (oo)cysts per field of view at 200x magnification as: 1+ (1–9 (oo)cysts), 2+ (10–50 (oo)cysts), or 3+ (51–100) (oo)cysts) or 4 + > 100 per field of view).

For samples classified as 2 + and above, DNA was extracted for molecular analysis.

### DNA extraction

DNA was extracted from samples scored as at least 2 + for at least one of the parasites using DNeasy PowerSoil DNA Isolation Kit (Qiagen), following the manufacturer’s protocol. In brief, 250 µL of faecal sample was homogenized in Power Bead tubes by bead beating using a FastPrep-24 5G (MP Biomedicals) with two cycles of 4 m/s for 60 s with a 45 s pause between the cycles. The DNA was collected on spin column filter membranes, before being washed and then eluted into 50 µL of the solution and stored at -20 °C until PCR was conducted.

### Polymerase chain reaction and sequencing

For *Cryptosporidium* species identification, primers targeting the small subunit *(SSU)rRNA* gene were used [13], and for subtyping *C. parvum* and *C. ubiquitum* primers targeting the *gp60* gene were used [14, 15]. For *Giardia* species/assemblage identification, primers targeting the beta-giardin (*BG*) gene was used [16]. All PCR reactions were carried out in a total volume of 25 µL that included 2 µL of template DNA, 1 µL forward and 1 µL reverse primer (0.4 µM concentration) and 12.5 µL of DreamTaq PCR Master Mix (2X) (Thermo Fisher Scientific) and 8.5 µL nuclease-free water.

Amplicons were separated by gel electrophoreses (2% agarose gel, stained with SYBERsafe DNA gel stain) and the results visualized under UV illumination. A 100 bp ready-to-use DNA ladder (Thermo Scientific) was used for fragment size determination. PCR products of the correct size were purified using ExoSAP-IT PCR product clean up (Thermofisher Scientific), then sent to a commercial facility in Germany (Eurofins Genomics) for sequencing. The sequences were examined and combined using Geneious Prime software, and the contig results compared with other sequences in GenBank using the NCBI BLAST tool. In addition, the online Cryptogenotyper tool [17] was used to investigate the various *Cryptosporidium* sequences obtained. Representative sequences were submitted to GenBank.

### Statistics

A database was created in Excel and analysed using STATA 15 software (STATA SE Corp, TX, USA) software. McNemar’s test was used to investigate the difference in shedding of *Cryptosporidium* oocysts and *Giardia* cysts by age (sampling occasion). Logistic regression analysis was used to assess the strength of association between *Cryptosporidium* and *Giardia* shedding and diarrhoea. Ordinal logistic regression was used to investigate the relationship between intensity of *Cryptosporidium* and *Giardia* shedding and diarrhoea. Mixed logistic regression was used with *Cryptosporidium* and *Giardia* with binary outcomes, location as explanatory variable and animal identification as a random variable. The confidence level was maintained at 95% and *P* < 0.05 was considered for the significance level.

## Results

### Occurrence of *Cryptosporidium* spp. and *Giardia* spp. in lambs from 17 Norwegian farms by age group

Of the 394 samples collected for analysis, 66 (17%) were positive for *Cryptosporidium* spp. and 61 (16%) were positive for *Giardia* spp. by IFAT (Table 1). Occurrence of *Cryptosporidium* and *Giardia* varied significantly by age group. For *Cryptosporidium*, significantly fewer lambs were shedding oocysts at the first sampling than at the second sampling (*P* < 0.001), or at the third sampling than at the first sampling (*P* < 0.001). However, samples taken on the second occasion had a significantly greater likelihood of containing *Cryptosporidium* oocysts than those taken at the third sampling. Occurrence of *Giardia* in the faecal samples also varied significantly by age group, with the lowest occurrence recorded at the first sampling and the highest at the third sampling. As with *Cryptosporidium*, the occurrence of *Giardia* was significantly lower in the first sampling than at the second sampling (*P* < 0.001) or at the third sampling (*P* < 0.001). However, the difference in occurrence between the second and third samplings was only of borderline significance for *Giardia* (*P* = 0.052).

**Table 1:** Occurrence of *Cryptosporidium* and *Giardia* in lamb samples by lamb age

Approximate age of sampled lambs	Total (*n*)	*Cryptosporidium* detected		*Giardia* detected		
		*n*	% (95% CI)		*n*	% (95% CI)
1 (2–3 days)	160	16	10.0 (6–15)		5	3.1 (1–7)
2 (14–21 days)	134	34	25.4 (18–33)		24	18.0 (12–25)
3 (around 42 days)	100	16	16.0 (9–24)		32	32.0 (23–41)
TOTAL	394	66	16.8 (13–20)		61	15.5 (12–19)

### *Cryptosporidium* and *Giardia* infection in lambs from Vestland and Rogaland

More samples were collected from Vestland (287) than from Rogaland (107) (Table 2).

Using mixed logistic regression analysis, *Cryptosporidium* infection was found in significantly more samples from Rogaland than Vestland (*P* < 0.004). However, the opposite pattern was seen for *Giardia* infection, with significantly more samples positive from Vestland than from Rogaland (*P* < 0.04). It should be noted, however, that this may reflect that relatively few samples were collected from Rogaland on the third sampling occasion, when most samples from Vestland were found to be *Giardia*-positive.

**Table 2:** Number of samples collected from Vestland and Rogaland by lamb age group

Location	Approximate age of sampled lambs			Total (*n*)
	2–3 days *n* (%)	14–21 days *n* (%)	40–42 days *n* (%)	
Vestland	108 (36)	92 (32)	87 (30)	287
Rogaland	52 (45)	42 (39)	13 (12)	107
**Total**	160 (39)	134 (34)	100 (27)	394

### Longitudinal investigation of infections

Although we did not obtain samples from all lambs at each sampling occasion, we attempted to investigate whether infected lambs retained their infection (or became reinfected), over a period of a few weeks, as summarised in Table 3.

**Table 3:** Changes in infection status of individual lambs at the individual sampling occasions

	Positive on all 3 sampling occasions	Positive on 1st and 2nd sampling occasions	Positive on 1st and 3rd sampling occasions	Positive on 2nd and 3rd sampling occasions
***Cryptosporidium***	0	3	0	4
***Giardia***	0	1	1	8

Of the 16 lambs shedding *Cryptosporidium* at the first sampling occasion, 3 (19%) were still positive at the 2nd sampling occasion around two weeks later. As four of the original positive lambs were not sampled on the second occasion, this indicates that there was continuation of infection, or repeated infection, in at least 25% of the lambs. At the final sampling occasion, approximately 3 weeks later, samples were collected from only 5 of the original 16 positive lambs, and none of these were shedding *Cryptosporidium*. Thus, none of these lambs were infected over the 42-day duration of sampling.

Of the 34 lambs shedding *Cryptosporidium* at the second sampling occasion, samples were collected from 23 at the final sampling occasion, and 4 were still shedding *Cryptosporidium* indicating continuation of infection or repeated infection in at least 18% of the lambs over this three-week period.

For *Giardia*, of the five lambs positive at the first sampling occasion, samples were obtained from four at the second sampling occasion and one of these was shedding *Giardia*, indicating continuation of infection or repeated infection in at least 25% of the lambs. At the final sampling, only one of the lambs that was positive at the first sampling was sampled again and was now positive – although it was also sampled on the second sampling occasion and *Giardia* cyst shedding had not been detected. At the second sampling occasion, 24 lambs were found to be shedding *Giardia* cysts, and 19 of these were sampled on the third occasion with 8 of these samples found to be positive. This indicates that around 42% of the lambs had either continued to be infected, or had been reinfected, during the intervening three weeks between the second and third sampling occasions.

### Association of *Cryptosporidium* and/or *Giardia* infection with diarrhoea in different age groups

Many of the lambs sampled in Vestland had diarrhoea, but only one lamb (the same lamb on two occasions) from Rogaland (Table 4).

**Table 4:** Occurrence of diarrhoea in lambs at the three sampling occasions in Rogaland and Vestland

Sampling occasion (approximate lamb age)	Number (%) of lambs with diarrhoea at sampling	
	Rogaland	Vestland
1st sampling (2–3 days)	1/52 (2)	46/108 (42)
2nd sampling (14–21 days)	1/42 (2)	43/92 (47)
3rd sampling (around 42 days)	0/13 (0)	43/87 (49)

Of the lambs that were classified as having diarrhoea at the last sampling, all except for one had also been recorded as having diarrhoea on the second sampling occasion, and all had also been recorded as having diarrhoea at the first sampling occasion.

On the second and third sampling occasions there was a significant association between *Cryptosporidium* shedding and diarrhoea, with *Cryptosporidium* detected in 41% of the diarrhoeic samples on the second occasion compared with 18% of the non-diarrhoeic samples (*P* < 0.005) collected at the same time as determined by logistic regression analysis. Similarly, *Cryptosporidium* was detected in 26% of the diarrhoeic samples on the third sampling occasion, but only in 9% of the non-diarrhoeic samples (*P* < 0.03) collected at the same time. Such an association was not detected on the first sampling occasion.

A similar pattern was seen for *Giardia*, with a significant association between shedding of *Giardia* cysts and diarrhoea on the second and third sampling occasions. *Giardia* was detected in 36% of the diarrhoeic samples compared with 9% of the non-diarrhoeic samples on the second occasion (*P* < 0.0002) and in 47% of the diarrhoeic samples on the third sampling occasion, compared with 21% of the non-diarrhoeic samples (*P* < 0.007). No association was found between *Giardia* infection and diarrhoea on the first sampling occasion.

### Intensity of infection

In general, the intensity of shedding *Cryptosporidium* and *Giardia* was low, with the majority of positive samples scoring just 1 + for both parasites. However, there was some variation by age group, the youngest age groups shedding only low numbers of parasites (no lambs scored as 3 + or 4+), and the highest shedding (+ 4) only observed in the oldest age group. Ordinal logistic regression analysis indicated a significant association between the intensity of shedding *Cryptosporidium* oocysts and *Giardia* cysts in those lambs that had diarrhoea (*P* < 0.05).

### Molecular characterisation

Due to low oocyst numbers, only 10 (16%) of the *Cryptosporidium*-positive samples were further analysed using molecular methods. Results were obtained for 8 of these with *C. ubiquitum* subtype XIIa identified in 3 samples from the same farm in Vestland (lambs’ ages 14–21 days) and *C. parvum* subtype IIaA13G1R2 identified in one farm in Rogaland (4 samples; lambs’ ages 14–21 days) and *C. parvum* subtype IIdA20G1 identified from one farm in Vestland (1 sample; lamb age 40–42 days). Although lambs shedding *C. ubiqutum* oocysts had diarrhoea, this was not the case for lambs infected with either of the subtypes of *C. parvum* identified. Representative GenBank submissions at the *Gp60* gene have the following Accession numbers: *C. ubiquitum* subtype XIIa (PQ540728), *C. parvum* subtype IIaA13G1R2 (PQ760435), *C. parvum* subtype IIdA20G1 (PQ540729).

Similarly, only 7 (12%) of the *Giardia*-positive samples were further analysed using molecular methods, with results only obtained from two of these (lambs’ ages: 14–21 days and 40–42 days old). Both these were found to be *G. bovis* (previously described as *G. duodenalis*, Assemblage E). A representative sequence of this isolate has been submitted to GenBank (Accession number: PQ540727).

For all sequences, except for *C. ubiquitum* subtype XIIa (PQ540728), there was 100% similarity to other sequences in GenBank with 100% coverage. For *C. ubiquitum* subtype XIIa (PQ540728), there was 100% identity with sequences in GenBank (e.g., KY711406.1 reporting this sequence from cattle in Ghana), but the coverage is only 92%, so that sequence is shorter.

## Discussion

The main finding of this study was that although both *Cryptosporidium* and *Giardia* infection were commonly found lambs of the three different ages in both study areas, their distributions across age groups varied and there were also regional variations.

Lambs from Rogaland had a greater occurrence of *Cryptosporidium* than those in Vestland, possibly due to larger flock size in the farms in this region, resulting in a higher infectious pressure as previously suggested as a risk factor [4]. Although *Giardia* occurrence appeared to be greater in Vestland than Rogaland, relatively few samples were obtained in the third round of sampling from Rogaland, when *Giardia* occurrence was highest in Rogaland and thus the data are skewed.

The highest occurrence of *Cryptosporidium* shedding was found when the lambs were sampled at around 14–21 days old; although sampling when the lambs were younger (2–3 days) identified that around 10% were shedding oocysts and had presumably been infected shortly after birth, the highest occurrence was found when the lambs were about 14–21 days old, then decreasing when they were around 42 days old, presumably reflecting onset of immunity. Some previous studies have indicated similar data (as summarised in [18]). A recent experimental *Cryptosporidium* infection study of lambs [12] indicated that the mean point at which faecal oocysts were first detected was around 6–7 days post-infection, with a standard deviation of around 3, suggesting that it is likely that many more of the lambs were infected at the first sampling point in our study (lambs − 2–3 days old), but oocyst shedding had not yet begun. It is possible that some of the oocysts detected in these younger lambs actually represented passage of non-infectious oocysts, rather than oocysts derived from sexual reproduction of the parasites following infection. However, it is also clear from outbreaks, as well as the experimental infection study [12], that some very young lambs may shed infectious oocysts within a day or so after infection.

From the longitudinal nature of our study, we were able to demonstrate that some lambs continued to shed oocysts after relatively prolonged periods, with oocysts detected on more than one sampling occasion (although none of the lambs sampled on all three occasions were found to be shedding on all occasions). The shedding on two sampling occasions could indicate either prolonged infection or reinfection. The experimental infection study in neonatal lambs [12] also demonstrated that infected lambs do not develop sterile immunity against re-infection with *Cryptosporidium*, and therefore re-infection cannot be excluded for those lambs that were shedding on more than one sampling occasion in our study. Unfortunately, we were unable to determine if the species or isolate of *Cryptosporidium* in lambs shedding oocysts on more than one occasion was the same or different; however, the work reported in [12] indicated that reinfection with the same species could occur.

Interestingly, among the few longitudinal studies published on these infections in lambs, one from Australia [19] indicated that the occurrence of *Cryptosporidium* in lambs on two farms, increased from 2 to 6 weeks old age to 2 months to 3–4 months old age, with a slight decline at 6–7 months old age. This could reflect that these researchers used a more sensitive molecular method for detection, and that we did not identify low-shedders. However, as oocyst shedding was not quantified in the Australian study [19], this is impossible to determine. As their study identified some lambs that shed oocysts on all 5 of their sampling occasions, either prolonged infection or re-infection was again suggested.

For *Giardia*, the proportion of lambs shedding cysts increased with age, rising steadily from relatively few at the youngest age group, to over 30% at the final sampling. This pattern of rising infection over time in lambs at pasture was identified over three decades ago [20]. Interestingly, however, although a longitudinal study [19] indicated a similar pattern, it was not so marked, with the occurrence of *Giardia* shedding in lambs at both farms in their study being between 20 and 30% of each of the five sampling occasions from 2 to 6 weeks until 7–9 months of age. Again, this could reflect differences in sampling and analysis approaches. As seen with *Cryptosporidium*, the longitudinal nature of our study demonstrated either prolonged infection and *Giardia* cyst shedding, or reinfection, with individual lambs positive for *Giardia* cysts on more than one occasion; chronic *Giardia* infection has been well documented and is unsurprising. The long-term effects on productivity and health of prolonged *Giardia* infection have not, to our knowledge, been investigated in sheep.

Infection was significantly associated with diarrhoea in our study, although not all lambs were diarrhoeic, and infection intensity was also relevant. The association of these intestinal protozoa with diarrhoeal disease is well established, particularly for *Cryptosporidium* infection [18], but less consistently for *Giardia* infection [21]. The studies investigating association of these infections with diarrhoea in farm settings must also take into account coinfection with other parasites, including *Eimeria* and nematodes, as well as infections with other agents [7, 18]. Asymptomatic carriage of both infections has previously been identified in lambs [22], and we also found high-shedding lambs with no overt symptoms.

Although we were unable to use molecular characterisation of the parasites to follow longitudinal trends, two species of *Cryptosporidium* were identified among our samples, *C. parvum* and *C. ubiquitum.* This was of particular interest, as a previous study in lambs at six different farms in Norway (2 in Rogaland, 1 in Oppland, and 3 in Hedmark) detected only *C. ubiquitum* (then known as cervine genotype) and *C. xiaoi* [7]. In that study, *C. xiaoi* was detected only in the younger lambs (5–6 weeks of age), but we did not identify it at all in the current study, despite investigating lambs of a similar age group. However, we did detect *C. parvum* (in lambs of 14–21 days age and around 42 days age), which was not found in any of the lambs of the 2010 study. In addition, we detected *C. ubiquitum* in the lambs of 14–21 days of age. A review article has suggested that *C. parvum* is the predominant species of *Cryptosporidium* infecting small ruminants [9]; however, this may be due to a predominance of studies from specific European countries (such as Italy, Spain, and UK), as well as the potential for incorrect data extraction (e.g., misreporting of *C. xiaoi* as *C. parvum*, as has been done in the review [9] of the data regarding the report from lambs in Norway [7]). Whereas some surveys of *Cryptosporidium* in sheep from other countries (e.g., Algeria) have reported similar results as we found, with a preponderance of *C. parvum* and with *C. ubiquitum* in second place [23], others (e.g., from USA) have reported data more similar to the earlier study from Norway, with a predominance of *C. xiaoi* [24].

Of the *C. parvum* subtypes identified in the current study (IIaA13G1R2 and IIdA20G1), one of these (IIdA20G1) was identified among human infections in Norway during 2022–2024 [25], and an outbreak of cryptosporidiosis in veterinary students in Norway in 2024 associated with lamb contact was due to a similar, but not identical, subtype of IIdA23G1 [10]. The IIaA13G1R2 subtype has not previously been identified in human infections in Norway, although similar genotypes (e.g., IIaA13G2R1, IIaA13G1R1) have been reported [25]. Interestingly, however, a case from UK describes children acquiring *Cryptosporidium* infection of subtype IIaA13G1R2 due to lamb contact [26].

*C. ubiquitum*, which was found in both this study and the previous study from Norway, has been recognized as an emerging zoonotic species [15] and is considered to be the most commonly identified species infecting sheep in North and South America [15]. The XIIa subtype, as identified in our study, has been reported to occur in around 90% of *Cryptosporidium* infections in sheep, and has been identified in various human infections in UK and USA [15]. In the survey of species of *Cryptosporidium* involved in human cases of cryptosporidiosis in Norway from 2022 to 2024 [25], among 359 isolates successfully speciated, only three were *C. ubiquitum*; for one of these, the infection was considered to have been acquired in Norway. In that study [25], *GP60* subtyping of *C. ubiquitum* isolates was not performed.

Although both species of *Cryptosporidium* identified in our current study are of public health significance, both being considered zoonotic, the *Giardia* that was molecularly characterised in our study was *G. bovis*. This species is considered to be host-adapted with little or no zoonotic potential [27]. This was also the *Giardia* species predominantly identified in the previous study from Norway [7], indicating that lambs are not likely to be a source of human *Giardia* infection. Nevertheless, use of a multilocus approach, in which several different genes were targeted by PCR, may have provided more extensive results and would be recommended for any future similar studies.

## Conclusions

This longitudinal study provided information on *Cryptosporidium* and *Giardia* infections in lambs on pasture in western Norway over a six-week time period. Both parasites occurred widely, although with some differences in regional and age distribution. Although the highest occurrence of *Cryptosporidium* oocyst shedding occurred when lambs were a couple of weeks old, it is noteworthy that a substantial proportion of lambs were shedding oocysts at only a couple of days of age, highlighting the potential for transmission even from the youngest lambs. The highest occurrence of *Giardia* cyst shedding was, as expected, at the last sampling, when the lambs were around 6 weeks old. Both infections were associated with diarrhoea, but several lambs without diarrhoea were also shedding these parasites, which also emphasizes the importance of realising that even non-diarrhoeic faeces may contain potential infectious parasites.

Longitudinal studies such as this one are relatively unusual, and our study reinforced the results of previous longitudinal investigations, that some lambs apparently retained these infections over a period of weeks (or became reinfected).

Molecular characterisation indicated that although those isolates of *Giardia* that were characterized were all *G. bovis*, which is not generally considered to have zoonotic potential, *Cryptosporidium* in lambs may be of public health relevance. Both *Cryptosporidium* species detected in lambs between 14 and 42 days of age, *C. parvum* and *C. ubiquitum*, are zoonotic, with *C. parvum* being of particular concern.

## Supplementary Information

Below is the link to the electronic supplementary material.

Supplementary Material 1

## Data Availability

The datasets used and/or analysed during the current study are available from the corresponding author on reasonable request. Representative sequences from different samples have been deposited in the public database GenBank, and the Accession numbers provided in the text.

## Declarations

### Prior publication

Some of the data included in this article have previously been published as an undergraduate thesis report. It is available via Brage, Norwegian University of Life Sciences 2022. https://nmbu.brage.unit.no/nmbu-xmlui/handle/11250/3029935.

## Abbreviations

IFAT: Immunofluorescent antibody test
mAbs: Monoclonal antibodies
DAPI: 4’6-diamidino-2-phenylindole
