# Supplementary material for: Cryptosporidium and Giardia infections of lambs in Southwest Norway: a longitudinal study
Source: Acta Vet Scand. 2025 Aug 13;67:40. doi: 10.1186/s13028-025-00823-8 (PMC12351989; doi:10.1186/s13028-025-00823-8)
Supplement: Supplementary file 1 — Supplementary Material 1 [file 13028_2025_823_MOESM1_ESM.pptx]

## Slide 1
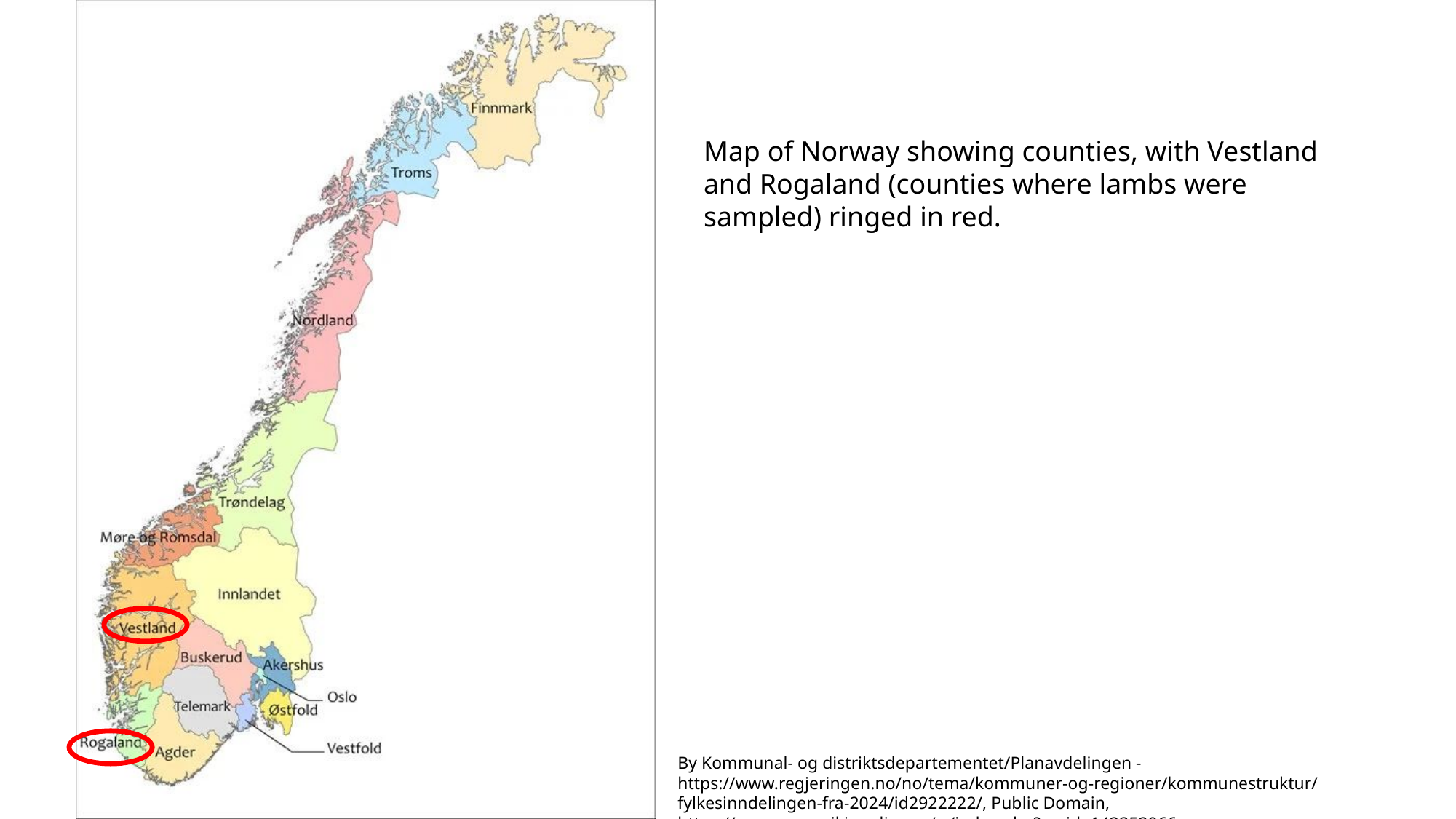

Map of Norway showing counties, with Vestland and Rogaland (counties where lambs were sampled) ringed in red.
By Kommunal- og distriktsdepartementet/Planavdelingen - https://www.regjeringen.no/no/tema/kommuner-og-regioner/kommunestruktur/fylkesinndelingen-fra-2024/id2922222/, Public Domain, https://commons.wikimedia.org/w/index.php?curid=143352966
